# Characterization of Class V DyP-Type Peroxidase SaDyP1 from *Streptomyces avermitilis* and Evaluation of SaDyPs Expression in Mycelium

**DOI:** 10.3390/ijms22168683

**Published:** 2021-08-12

**Authors:** Kanako Sugawara, Toru Yoshida, Rena Hirashima, Ryoko Toriumi, Hotaka Akiyama, Yurika Kakuta, Yuki Ishige, Yasushi Sugano

**Affiliations:** 1 Department of Chemical and Biological Sciences, Faculty of Science, Japan Women’s University, 2-8-1 Mejirodai, Bunkyo-Ku, Tokyo 112-8681, Japan; sugawarak@fc.jwu.ac.jp (K.S.); yoshidat@fc.jwu.ac.jp (T.Y.); hirashima.jw.tit@gmail.com (R.H.); m1617062tr@ug.jwu.ac.jp (R.T.); hotakaakiyama@icloud.com (H.A.); kakutay999@gmail.com (Y.K.); tiramisu_g5t5a3t2kitty@yahoo.co.jp (Y.I.); 2 Department of Clinical Laboratory Sciences, Faculty of Health Sciences, Nihon Institute of Medical Science, 1276 Shimogawara, Moroyamamachi, Irumagun, Saitama 350-0435, Japan

**Keywords:** heme peroxidase, dye-decolorizing peroxidase, DyP-type peroxidase, *Streptomyces*

## Abstract

DyP-type peroxidases are a family of heme peroxidases named for their ability to degrade persistent anthraquinone dyes. DyP-type peroxidases are subclassified into three classes: classes P, I and V. Based on its genome sequence, *Streptomyces avermitilis*, eubacteria, has two genes presumed to encode class V DyP-type peroxidases and two class I genes. We have previously shown that ectopically expressed SaDyP2, a member of class V, indeed has the characteristics of a DyP-type peroxidase. In this study, we analyzed SaDyP1, a member of the same class V as SaDyP2. SaDyP1 showed high amino acid sequence identity to SaDyP2, retaining a conserved GXXDG motif and catalytic aspartate. SaDyP1 degraded anthraquinone dyes, which are specific substrates of DyP-type peroxidases but not azo dyes. In addition to such substrate specificity, SaDyP1 showed other features of DyP-type peroxidases, such as low optimal pH. Furthermore, immunoblotting using an anti-SaDyP2 polyclonal antibody revealed that SaDyP1 and/or SaDyP2 is expressed in mycelia of wild-type *S. avermitilis*.

## 1. Introduction

Heme peroxidases are conserved among almost all organisms and catalyze various oxidative reactions using hydrogen peroxide as an electron acceptor. They are present in both prokaryotes and eukaryotes and are classified by the peroxidase and oxide-reductase database RedoxiBase (https://peroxibase.toulouse.inra.fr/, accessed on 20 July 2021) into six groups [1,2]: catalases, haloperoxidases, di-heme cytochrome c peroxidases, animal peroxidases, non-animal peroxidases (previously called plant peroxidase [3]) and DyP-type peroxidases. Dye-decolorizing peroxidase (DyP; EC 1.11.1.19), a novel peroxidase isolated from the basidiomycete *Bjerkandera adusta* Dec 1 [4], is capable of degrading persistent anthraquinone dyes, in contrast with other types of peroxidases [5]. DyP differs from existing heme peroxidases in terms of amino acid sequence, tertiary structure, substrate specificity, catalytic residues and optimum pH. For these reasons, it was originally considered an exceptional peroxidase. However, DyP and a series of genes phylogenetically similar to DyP were proposed to constitute a novel heme peroxidase family, the DyP-type peroxidase because many genes similar to DyP were found, and for those cases in which the corresponding protein has been purified, their biochemical characterizations have been revealed similar to those of DyP [6,7].

Conventionally, RedoxiBase has subdivided DyP-type peroxidases into classes A, B, C and D based on similarity of amino acid sequences [1]. However, although amino acid sequences between different classes exhibit low similarity, tertiary structures are highly similar. Therefore, a new classification of the DyP-type peroxidase family has been proposed using structure-based sequence alignments [8]. In this new scheme, classes P and I correspond to former classes B and A, respectively, whereas former classes C and D are combined into a new class V.

Many enzymes have been annotated as DyP-type peroxidases and characterized based on expression and biochemical properties of purified proteins, establishing DyP-type peroxidases as enzymes with potentially very diverse but still poorly physiological functions. The full range of DyP-type peroxidase roles remains unclear, but it is increasingly thought that these functions may be subclass dependent. Most DyPs from basidiomycetes belong to class V. These include TAP from *Termitomyces albuminosus* [9], AjPI and AjPII from *Auricularia auricula-judae* [10], MsP1 and MsP2 from *Marasmius scorodonius* [11], DyP-type peroxidase from *Irpex lacteus* [12], *Mep*DyP from *Mycena epipterygia* [13] and *Egl*DyP from *Exidia glandulosa* [13], all of which can degrade a lignin, suggesting that they have physiological roles in lignin degradation. We have also recently shown that antimicrobial substances retained by trees that prevent parasitism from fungi are candidate physiological substrates of DyP from *B. adusta* Dec 1 [14]. However, research on class V enzymes from bacteria is limited to DyP2 from *Amycolatopsis* sp. [15], AnaPX from *Anabaena* sp. [16,17,18] and SaDyP2 from *S. avermitilis* [19]. The physiological roles of them have not yet been established. Most bacterial DyP-type peroxidases belong to classes P and I, and are not limited to oxidative degradation of substrates, being capable of performing various functions in the cell. For instance, YfeX and EfeB (YcdB) from *Escherichia coli* may function as dechelatases, extracting an iron atom from heme without causing tetrapyrrole degradation [20]. However, whether YfeX and EfeB actually function as dechelatases *in vivo* remains unclear [21]. Furthermore, EfeB is also a component of a ferrous iron transporter [22], and it has been reported that several DyPs are likely to function in iron transport [23,24,25].

*Streptomyces avermitilis* is a gram-positive eubacterium whose entire genome has been analyzed [26]. *S. avermitilis* has four genes that are predicted to encode proteins with amino acid sequences highly similar to those of DyP-type peroxidases. Two of them, *SavDyPrx03-1* (SAV_549) and *SavDyPrx03-2* (SAV_3599), are deposited as class C DyP-type peroxidases (now class V) in RedoxiBase [1,2]. Using ectopic expression and biochemical characterization, we previously showed that SaDyP2, encoded by *SavDyPrx03-2*, indeed has features of a DyP-type peroxidase. The remaining two genes, SAV_4242 and SAV_5925, encode enzymes that are predicted to be class A (now class I) DyP-type peroxidases based on a Peroxiscan search in RedoxiBase [1,2]. In the case of SAV_4242, its homolog in *S. lividans*, DtpA, was reported to function in the formation of aerial mycelium via copper transport [27]. In addition to its function, the crystal structure and enzymatic reaction mechanism of DtpA have been investigated in detail [28,29,30,31]. Crystal structures of the enzyme from *S. coelicolor* have also been registered in the Protein Data Bank (PDB: 4GRC and 4GT2). However, virtually all studies in *Streptomyces* to date have been on Class I enzymes, with few studies on Class V enzymes. In addition, although *S. lividans* and *S. coelicolor* are well-studied *Streptomyces*, the genes encoding class V DyP-type peroxidases have not been found in either species. In this study, we ectopically expressed the DyP-type peroxidase encoded by *SavDyPrx03-1* (SAV_549), termed SaDyP1, and characterized it. In addition, immunoblotting demonstrated the expression of class V enzymes in wild-type *S. avermitilis*.

## 2. Results

### 2.1. Amino Acid Sequences and Structure Alignment

**Figure 1:**
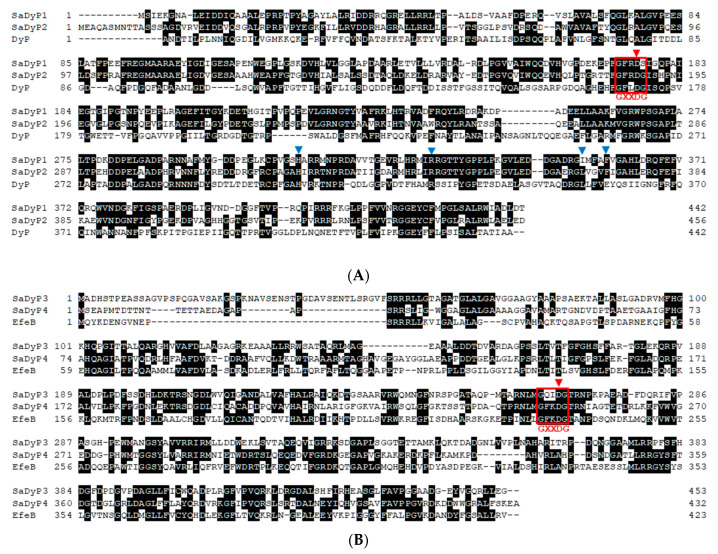

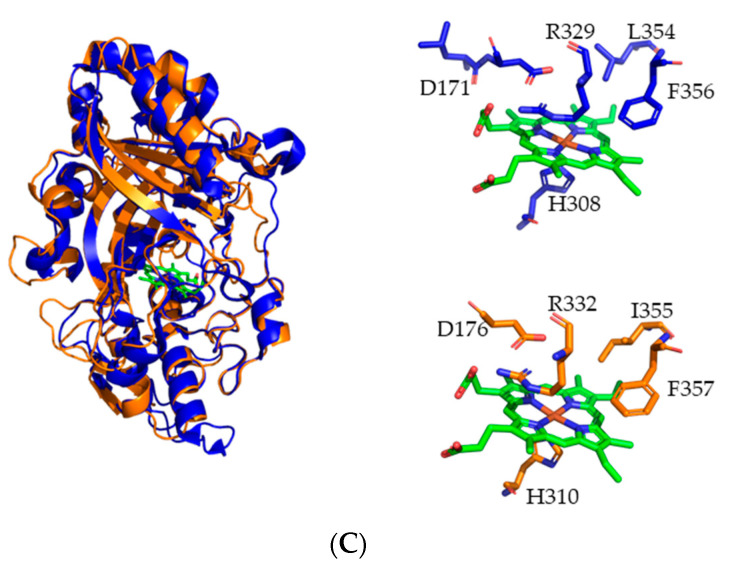
Amino acid sequence and structure alignments between SaDyPs and representative DyP-type peroxidases. (**A**) SaDyP1, SaDyP2 and DyP from *B. adusta*. (**B**) SaDyP3, SaDyP4 and EfeB. Residues with two or more matches are indicated with black fill. The unique GXXDG motifs of DyP-type peroxidases are surrounded by a red line. Catalytic aspartate of DyP is indicated by red arrowheads [6]. Blue arrowheads show four amino acid residues that form the hydrogen peroxide binding pocket of DyP [21]. (**C**) Homology modeling of SaDyP1 using SWISS-MODEL. Orange, the model of SaDyP1; blue, DyP. A model constructed using an ancestral class V DyP-type peroxidase (PDB:7anv) as a template. Left, structure alignment of the model of SaDyP1 and DyP (PDB: 3afv). Upper-right, enlarged view around heme in DyP (PDB: 3afv). Catalytic aspartate and four amino acid residues highly conserved around heme in DyP-type peroxidase were indicated. Bottom, enlarged view around heme in the model of SaDyP1. Amino acid residues of SaDyP1 model corresponding in DyP (upper-right) were shown. The drawing and alignment were by the PyMOL Molecular Graphics System, Version 2.5 Schrödinger, LLC.

Whole-genome sequencing showed that *S. avermitilis* has four candidate DyP-type peroxidase genes [26]. Two of these, *SavDyPr03-1* (SAV_549) and *SavDyPrx03-2* (SAV_3599), encode proteins predicted to be class V (formerly class C in RedoxiBase) that we named SaDyP1 and SaDyP2, respectively [19]. A ClustalW-based amino acid sequence alignment of SaDyP1, SaDyP2 and DyP from *B. adusta*, a class V DyP-type peroxidase, is shown in Figure 1A. The amino acid sequences of SaDyP1 and SaDyP2 are quite similar, exhibiting 59.1% identity. On the other hand, the sequence identity with DyP is lower for SaDyP1 (27.2%) and SaDyP2 (28.3%). The remaining two genes, SAV_4242 and SAV_5925, are predicted to encode class I (formerly class A) proteins that we named SaDyP3 and SaDyP4, respectively. The amino acid sequence alignments of SaDyP3 and SaDyP4 with EfeB from *E. coli*, a Class I DyP-type peroxidase, are shown in Figure 1B. The amino acid sequence homology between SaDyP3 and SaDyP4 is 39.7%. SaDyP3 is a homologue of DtpA, a DyP-type peroxidase from *S. lividans*, and the two peroxidases show an amino acid sequence identity of 78.4%. The DyP-type peroxidase from *Streptomyces coelicolor* (PDB: 4GRC) is a SaDyP4 homologue, and these two enzymes exhibit an amino acid sequence identity of 85.6%. On the other hand, genes homologous to those encoding SaDyP1 and SaDyP2 do not exist in well-studied *Streptomyces* sp. such as *S. lividans* and *S. coelicolor*. Structure alignment of SaDyP1 model constructed by SWISS-MODEL and DyP (PDB: 3afv) was shown in Figure 1C. SaDyP1 has conserved aspartate D176, other conserved residues and unique GXXDG motif of DyP-type peroxidases. Comparison of the conserved amino acid residues around the heme, the positions of the residues were in reasonable correspondence between DyP and SaDyP1 models.

### 2.2. Purification of SaDyP1

**Figure 2:**
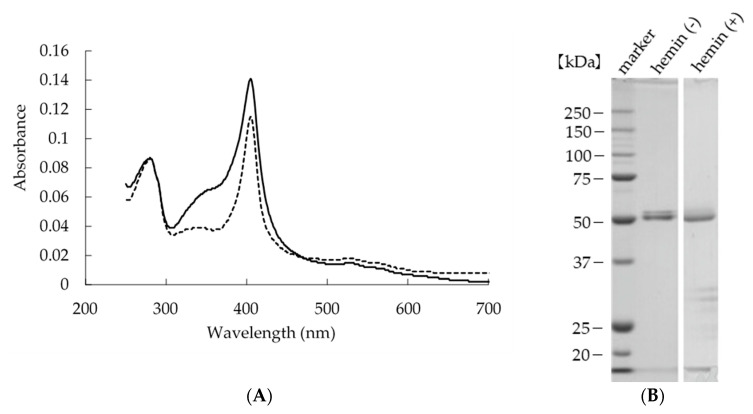
Analysis of purified SaDyP1, with or without hemin. (**A**) Electronic absorption spectroscopic characterization of purified SaDyP1, with or without hemin. Solid line, with hemin; dashed line, without hemin. A Soret band was observed at 406 nm. (**B**) SDS-PAGE analysis of purified SaDyP1, with or without hemin, stained with CBB. SaDyP1 was resolved by electrophoresis on a 10% polyacrylamide gel and then stained with CBB R-250.

We previously characterized heterologously expressed SaDyP2 using *S. lividans* as an expression host [19]. In the current study, we expressed and characterized SaDyP1 in the same manner. SaDyP1, similar to SaDyP2, was not secreted outside of cells [19]. Heterologously expressed heme peroxidases often adopt the apo form, which lacks a heme molecule, but the spectrum of expressed SaDyP1 showed apparent absorbance at 406 nm (Soret band), indicating incorporation of a heme molecule (Table 1 and Figure 2A). However, Reinheit Zahl (Rz) values suggested the presence of some apo form; thus, we incubated the Ni-NTA eluate with hemin chloride to incorporate heme. After dialysis and size-exclusion chromatography to remove free hemin and non-target protein, we characterized the spectrum and measured peroxidase activity. These analyses showed an increase in Rz value upon addition of hemin chloride but no increase in peroxidase activity (Table 1); the spectrum of SaDyP1 purified after adding the hemin reagent showed the presence of a shoulder peak around 350 nm (Figure 2A).

Purified SaDyP1 was resolved by sodium dodecyl sulfate-polyacrylamide gel electrophoresis (SDS-PAGE) and stained with Coomassie brilliant blue (CBB). Two bands appeared nearly 49.4 kDa, the predicted molecular mass of SaDyP1 with a 6 × His tag (Figure 2B). The N-terminal amino acid sequence of both bands contained the N-terminus of the SaDyP1 sequence. Therefore, the two bands are both derived from ectopically expressed SaDyP1. On the basis of these observations, we chose to use the SaDyP1 purified without hemin chloride for subsequent characterizations.

### 2.3. Optimal pH and Thermostability of SaDyP1

**Figure 3:**
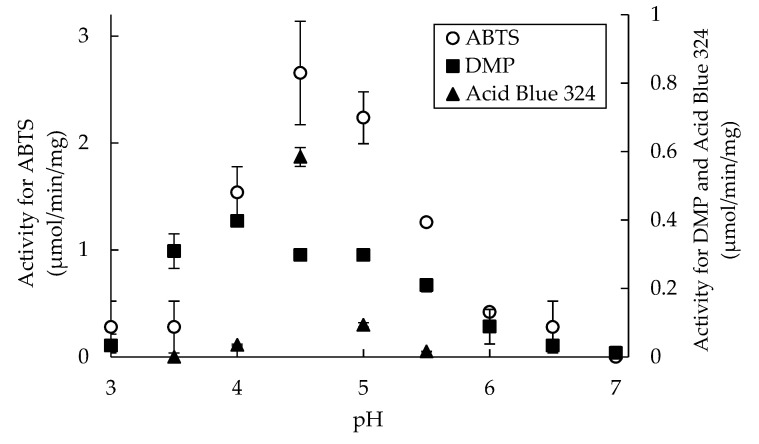
Optimal pH for the peroxidase activity of SaDyP1. Peroxidase activities against ABTS and DMP were measured between pH 3 and 7. Decolorizing activities against Acid Blue 324 were measured between pH 3.5 and 5.5 (*n* = 3). Open circle, ABTS; closed square, DMP; closed triangles, Acid Blue 324.

DyP-type peroxidase is characterized by a lower pH optimum than other heme peroxidase [6,32]. To determine whether SaDyP1 has a lower optimum pH, we measured peroxidase activity against ABTS (2,2′-azinobis [3-ethylbenzothiazoline-6-sulfonic acid]) and 2,6-dimethoxyphenol (DMP) between pH 3.0 and 7.0. SaDyP1 showed the highest peroxidase activity for ABTS at pH 4.5 and for DMP at pH 4.0 (Figure 3), essentially the same behavior as that shown by SaDyP2 [19]. To examine pH profile for anthraquinone dye, peroxidase activity against Acid Blue 324 was also measured between pH 3.5 and 5.5. SaDyP1 showed the highest activity for Acid Blue 324 at pH 4.5. Differently from those for ABTS and DMP, the degradative activity of SaDyP1 for Acid Blue 324 was almost undetectable, except at pH 4.5.

**Figure 4:**
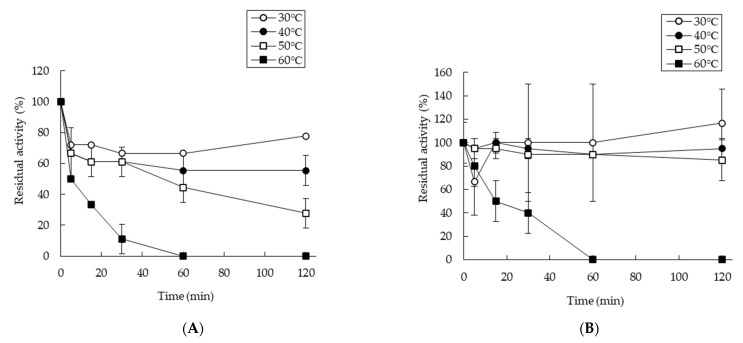
Thermostability of purified SaDyP1. SaDyP1 peroxidase activity toward ABTS (**A**) and DMP (**B**) at 30 °C after incubation at 30 °C, 40 °C, 50 °C or 60°C (*n* = 3). Residual activity at 0 h was defined as 100%. Open circles, 30 °C; closed circles, 40 °C; open squares, 50 °C; closed squares, 60 °C.

The thermostability of SaDyP1 activity was measured at 30 °C after treatment at various temperatures (Figure 4A,B). SaDyP1 retained almost all of its initial activity against ABTS and DMP after incubation at 30 °C or 40 °C for 2 h (Figure 4A,B). The peroxidase activity of SaDyP1 against ABTS significantly decreased after incubating at 50 °C for 1 h and was almost completely lost following incubation at 60 °C for 30 min (Figure 4A). On the other hand, the activity of SaDyP1 against DMP was maintained at 50 °C.

### 2.4. Substrate Specificity and Catalytic Properties

Class V DyP-type peroxidases are characterized by their ability to efficiently decolorize anthraquinone dyes, but not azo dyes. SaDyP1 is predicted to be a class V enzyme (formerly class C) based on amino acid sequence homology [1,2]. To assess the selectivity of the decolorizing activity of SaDyP1, we tested several anthraquinone and azo dyes as substrates. SaDyP1 decolorized the anthraquinone dye Acid Blue 324, but was inactive against the anthraquinone dyes AQ-2, M303 and all azo dyes tested (Table 2). In addition, the decolorizing activity against Acid Blue 324 was lower than that of DyP, which belongs to the same class V [33]. These results indicate that SaDyP1 belongs to class V, based on its degradative activity against anthraquinone dyes, although its activity is lower than that of most class V DyP-type peroxidases.

**Figure 5:**
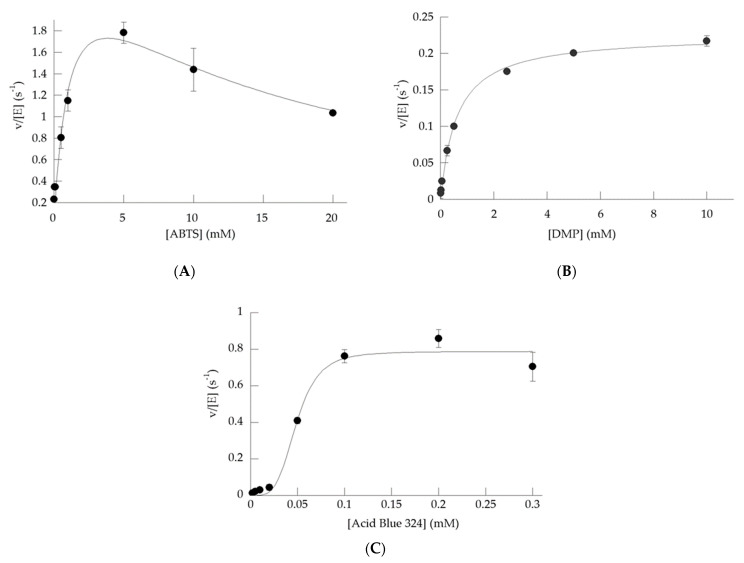
Substrate saturation curves of SaDyP1 for (**A**) ABTS at pH 4.5, (**B**) DMP at pH 4.0 and (**C**) Acid Blue 324 at pH 4.5 (*n* = 3). The plots were fitted to Haldane equation (ABTS), Michaelis-Menten equation (DMP) and Hill equation (Acid Blue 324).

To determine the kinetic parameters of SaDyP1 against ABTS, DMP and Acid Blue 324, we determined substrate saturation curves for these typical peroxidase substrates (Figure 5). The peroxidase activity of SaDyP1 toward ABTS increased in a substrate concentration-dependent manner up to 5 mM but decreased above 10 mM probably with substrate inhibition. Therefore, we fitted the activity for ABTS to the Haldane equation describing substrate inhibition kinetics [34] and calculated *k*_cat_, *K*_m_ and *K*_i_ (Table 3). The concentration-dependent activities with DMP were fitted to the Michaelis-Menten curve and the values of *k*_cat_ and *K*_m_ were calculated (Table 3). Then, concentration-dependent activity of Acid Blue 324, anthraquinone substrate, was plotted and a sigmoidal curve fitted to Hill equation was obtained (Hill coefficient = 4.1). The *K*_m_ values of SaDyP1 are similar to those previously reported for *B. adusta* DyP against ABTS and DMP [35]. In contrast, the *k*_cat_ values of SaDyP1 for ABTS and DMP were much lower than those for DyP. These results suggest that SaDyP1 is less capable of oxidizing typical peroxidase substrates than other class V enzymes.

### 2.5. Expression of SaDyPs in Wild-Type S. avermitilis Mycelia

**Figure 6:**
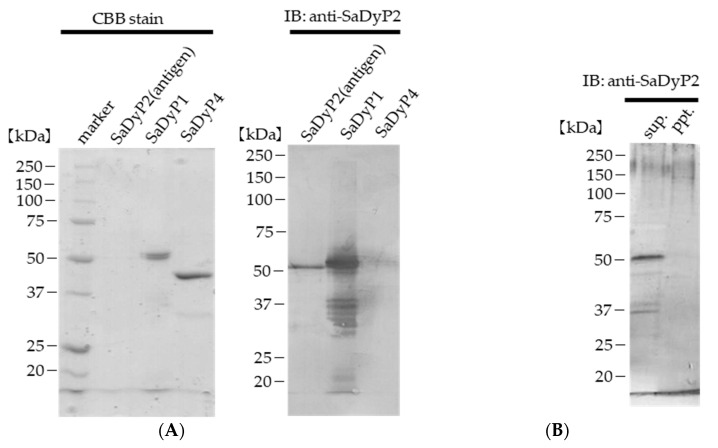
Cross-reactivity of an anti-SaDyP2 polyclonal antibody toward recombinant SaDyP1 and SaDyP4. (**A**) SDS-PAGE of SaDyP2 (antigen), SaDyP1 and SaDyP4, showing staining with CBB-G250 (left) and immunoreactivity to the anti-SaDyP2 antibody. (**B**) SDS-PAGE of *S. avermitilis* mycelium followed by immunostaining with anti-SaDyP2. sup., supernatant; ppt., precipitate.

To determine whether SaDyP is expressed in wild-type *S. avermitilis*, we performed immunoblotting using a polyclonal antibody against SaDyP2, generated using a purified SaDyP2 that was previously ectopically expressed in *S. lividans*. First, we examined the reactivity of the anti-SaDyP2 antibody against recombinant SaDyP1 expressed in *S. lividans* and SaDyP4 expressed in *E. coli*. SaDyP1 cross-reacted with anti-SaDyP2, but not SaDyP4 (Figure 6A, right), an outcome that is not entirely surprising given the high amino acid sequence homology between SaDyP1 and SaDyP2 (Figure 1A). Next, cultured mycelia of wild-type *S. avermitilis* were disrupted, and proteins in the resulting supernatant were resolved by SDS-PAGE and immunoblotted using a SaDyP2 antibody. This immunoblot analysis revealed SaDyP2 antibody-reactive band (Figure 6B). Given the cross-reactivity shown in Figure 6A and the molecular weight estimated from the mobility on SDS-PAGE gels, the observed bands can be presumed to be SaDyP1 and/or SaDyP2. These results indicate that at least one of these class V SaDyPs is indeed expressed in *S. avermitilis*.

## 3. Discussion

*S. avermitilis* is predicted to have four genes encoding DyP-type peroxidases. Since most bacterial DyP-type peroxidases are classified as classes P or I, SaDyP1 and SaDyP2 are expected to exert unique functions in *S. avermitilis*. SaDyP3 and SaDyP4 are predicted to belong to class I based on assignment to former class A by Peroxiscan, a program that facilitates the subclassification of amino acid sequences in the RedoxiBase. DtpA, a homologue of SaDyP3 in *S. lividans*, was recently proposed to function in the copper-dependent morphogenesis pathway [27]. SaDyP4 is very similar in amino acid sequence to EfeB (Figure 1A). On the other hand, the functions and characteristics of the homologs of SaDyP1 and SaDyP2 have not been reported. A BlastP search revealed that *Streptomyces hirsutus* contains an enzyme that is 73.0% identical in amino acid sequence to SaDyP1, and enzymes similar to SaDyP2 are found in several *Streptomyces* species, including *Streptomyces mirabilis* (95.3% identity), *Streptomyces olivochromogenes* (93.8%) and *S. hirsutus* (93.2%). However, compared with these *Streptomyces*, for which the few reports of gene disruption, a gene disruption method in *S. avermitlis* has been established [36], being suitable for clarifying the physiological function of class V DyP peroxidases from *Streptomyces.*

DyP peroxidase lack the distal histidine as an acid-base catalyst, and we have previously proposed that DyP utilizes aspartate instead [6,32]. The alignment of the amino acid sequences and structures of SaDyP1, SaDyP2 and DyP shown in Figure 1A,C showed that the distal aspartate and GXXDG motif are conserved in SaDyP1. However, Mendes et al. reported that the aspartate and arginine are important for the proper binding of H_2_O_2_ to the heme, but none is individually indispensable for promoting H_2_O_2_ (de)protonation and O-O bond cleavage in BsDyP from *Bacillus subtilis* [37]. D235A and R347E mutations in EfeB from *Escherichia coli* O157 inactivated the enzyme, but D235N retained its activity [38]. In DyPB from *Rhodococcus jostii* RHA1 and PpDyP from *Pseudomonas putida* MET94, the substitution of distal arginine, not of aspartate, results in a decrease in their activities [39,40]. Thus, it is a controversial issue, which residue functions as an acid-base catalyst in DyP-type peroxidase.

In most cases of recombinant expression of hemoproteins using *E. coli* as a host, the expressed proteins lack the cofactor heme. In these situations, heme is incorporated by adding 5-aminolevulinic acid, a precursor of heme, to the culture medium or by adding hemin chloride in vitro after expression. On the other hand, when we used *Aspergillus oryzae* as a host for the recombinant expression of DyP, the holoenzyme was expressed without any additional manipulation [5]. In the current study, a part of SaDyP1 recombinantly expressed in *S. lividans* as a host incorporated heme without any extrinsic addition and showed peroxidase activity (Table 1 and Figure 3). On the other hand, the Rz value of SaDyP1 increased upon addition of hemin chloride, suggesting that a percentage of apoenzymes incorporate heme. Interestingly, the peroxidase activity did not increase with the addition of hemin chloride, despite evidence of additional incorporation of heme into the apoenzyme. We hypothesized two reasons: (1) Free hemin, which could not be removed by dialysis or ultrafiltration, causing an apparent increase in Rz value and the additional peak at 350 nm. (2) Heme was incorporated into the inappropriate site of SaDyP1, causing the shoulder peak. In any case, since the addition of hemin did not increase the peroxidase activity, purification without hemin was adopted in this study. Since the expression efficiency of the pHSA81 vector and *S. lividans* expression system was comparable to that of the *E. coli* expression system, the observation that the *S. lividans* system incorporated much more heme than the *E. coli* system was unexpected. This greater efficiency of the *S. lividans* system, despite ectopic expression in both cases, may be attributable to the fact that the target protein and the expression host are of the same species. There are few reports for recombinant expression of heme proteins by pHSA81, and the incorporation of heme into heme proteins in ectopically expression is still under investigation.

SaDyP1 degraded the anthraquinone dye Acid Blue 324 but was inactive against the anthraquinone dyes AQ-2, M303 and all azo dyes tested (Table 2). These results are consistent with the unique features of DyP-type peroxidase, which is capable of degrading persistent anthraquinone compounds. *k*_cat_/*K*_m_ values of SaDyP1 for Acid Blue 324 were 1.61 × 10^3^ M^−1^s^−1^ (Figure 5C and Table 3). SaDyP2 exhibited *k*_cat_/*K*_m_ value of 1.24 M^−1^s^−1^ for Acid Blue 324 in our previous report [19]. By comparison, DyP and AjPI from *A. auricula-judae* showed *k*_cat_/*K*_m_ values of 10^7^ and 10^6^, respectively [8,10]. AnaPX from *Anabaena* sp. and DyP2 from *Amycolatopsis* sp. 75iv2—two prokaryotic enzymes belonging to class V—exhibited *k*_cat_/*K*_m_ values of 10^7^ and 10^5^ M^−1^s^−1^, respectively [15,17]. These results showed that the *k*_cat_/*K*_m_ of SaDyP1 and SaDyP2 for anthraquinone compounds were low. On the other hand, the *K*_m_ values of SaDyP1 and SaDyP2 for Acid Blue 324 were similar to those of *B. adusta* DyP. Thus, the lower *k*_cat_/*K*_m_ of SaDyP1 and SaDyP2 was accounted for the lower *k*_cat_. Even though a class I enzyme, the *k*_cat_ value of DtpA from *S. lividans* for Reactive Blue 19 was on the order of 10^−2^ to 10^−1^ estimated from the previous study [29], which is lower than that of other Class I enzymes such as TfuDyP (*k*_cat_ = 10 s^−1^), DyPA (*k*_cat_ = 13 s^−1^), BsDyP (*k*_cat_ = 7.9 s^−1^) [40,41,42]. As a reason why DyPs from *Streptomyces* show low degrading activities of some anthraquinone compounds is unknown, clarifying the basis for differences in decolorizing activities among enzymes belonging to class V will require more studies on class V enzymes from both prokaryotes and eukaryotes. One of the most powerful tools for such investigations is tertiary structural analysis, which enables a detailed discussion of the active site. Therefore, we consider the determination of the tertiary structure of SaDyP1 as a future challenge. 

DyP-type peroxidase is characterized by a lower pH optimum than other heme peroxidases. In this study, SaDyP1 actually showed a low pH optimum. It has been thought that the low p*K*_a_ value of aspartate contributes to the low pH optimum [6,32]. However, the p*K*_a_ value of amino acid residue buried into the protein is generally different from the p*K*_a_ value of free amino acid. Recently, Uchida et al. reported that radical transfer but not aspartate is essential for the low pH optimum [43]. A hydrogen bond located on a potential radical transfer route is responsible for the low pH optimum. The optimal pH values for SaDyP1 peroxidase activity against ABTS and DMP—pH 4.5 and 4.0, respectively—were exactly the same as those for SaDyP2 (Figure 3). These pH optima for SaDyP1 and SaDyP2 are slightly higher than the optimal pH for DyP (pH 3.2). SaDyP1 was thermostable at higher temperatures, retaining ~30% or more of its initial activity against ABTS and DMP even after incubation at 50 °C for 2 h. DyP has been reported to be thermostable compared with horseradish peroxidase [4], and its stability is contributed by N-linked glycosylations at three Asn residues located on DyP surface. On the other hand, it is unlikely that SaDyP1 from the prokaryote *S. avermitilis* is glycosylated, so the basis for the stability of SaDyP1 at 50 °C remains uncertain. Four genes from the genome of *S. avermitilis* have been annotated as DyP-type peroxidases, but to date it remains unclear whether all four are actually expressed. In this study, we observed a SDS-PAGE band that cross-reacted with anti-SaDyP2 antibody in the supernatant obtained from fragmented mycelia of *S. avermitilis*. In future, we will perform phenotypic studies following disruption of genes encoding SaDyP1 and SaDyP2 to clarify the physiological substrates and functions of these enzymes. 

In conclusion, we heterologously expressed and characterized SaDyP1 from *S. avermitilis*. We hypothesize that SaDyP1 has a putative aspartate residue responsible for catalysis based on sequence and structure alignments. Moreover, SaDyP1 shows a lower optimal pH, suggesting membership in the DyP-type peroxidase family. Typical substrates of peroxidases and anthraquinone dyes were oxidized by SaDyP1, although decolorizing rates were lower than those for DyP. Even though the results of this study demonstrated the expression of class V DyP-type peroxidases in *S. avermitilis*, there are no homologs of these proteins whose physiological substrates, functions, and tertiary structures have been elucidated; therefore, further study is warranted.

## 4. Materials and Methods

### 4.1. Organisms and Materials

*S. avermitilis* ATCC31267 was obtained from American Type Culture Collection (ATCC). pHSA81 and *S. lividans* were kindly provided by Dr. M. Kobayashi, University of Tsukuba. All chemical reagents and media used were analytical grade, unless otherwise specified.

### 4.2. Plasmid Construction and S. lividans Transformation

Plasmid construction and transformation procedures for expression of SaDyP1 in *S. lividans* were performed as previously described [19]. The gene encoding SaDyP1 was PCR-amplified from *S. avermitilis* genomic DNA using the primer pair 5′-GGA ATT CCA TAT G AGC ATC GAG AAG GGC-3′ (forward) and 5′-GGA CTA GTT CAG TGG TGA TGG TGA TGA TGG GTG TCG AGG TCG GCG ATC CAG CGC AGC GCC GAC AGA CCG G-3′ (reverse), and cloned as a 6 × His-tagged protein into the pHSA81 expression vector for *S. lividans*. Each primer included a restriction enzyme site to facilitate cloning. *S. lividans* was transformed with pHSA81 harboring the amplified gene using polyethylene glycol (PEG)-mediated transformation of protoplasts, after which antibiotic-resistant transformants were selected by culturing with 1 μg/mL thiostrepton. 

### 4.3. Purification of SaDyP1

Transformed cells were cultured in 100 mL YEME medium (0.3% yeast extract, 0.5% Bacto-Tryptone, 0.3% malt extract, 1% glucose, 0.5% glycine, 5 mM MgCl_2_, 34% sucrose, pH 7.0) at 29 °C for 3 days. Cells were disrupted, and soluble protein in the supernatant was purified by Ni-NTA agarose affinity chromatography (Qiagen, Hilden, Germany) [19]. The eluted fraction was dialyzed against 25 mM citrate buffer (pH 5.5). In cases where addition of hemin chloride was necessary, hemin chloride was added at three molar equivalents relative to approximate protein content after dialysis. Following overnight incubation to incorporate heme, the solution containing the protein was dialyzed again to remove unincorporated hemin and applied to a Superdex 75pg column (bed volume, 120 mL; Cytiva, Tokyo, Japan). Fractions (1 mL) were collected, and fractions containing enzyme activity were pooled.

### 4.4. Polyacrylamide Gel Electrophoresis, Immunoblotting and Spectral Characterization

SDS-PAGE was performed as previously described [19]. After electrophoresis, proteins in gels were either stained with Coomassie Brilliant Blue G-250 (CBB-stain) or transferred to membranes for immunoblotting using polyclonal antibodies, produced against previously purified SaDyP2, used as an antigen. Spectral characteristics of purified SaDyP1 in 25 mM citrate buffer (pH 5.5) were assessed using a V-650 UV-Vis spectrophotometer (Jasco, Tokyo, Japan). Reinheit Zahl (Rz) value was calculated as *A_406_/A_280_* ratio.

### 4.5. Enzyme Assays and Characterization

The optimum pH and thermostability of SaDyP1 were determined as previously described [19]. Briefly, SaDyP2 activities for ABTS, DMP and Acid Blue 324 were measured using a citrate buffer (25 mM) covering a pH range of 3.0–5.5 and a phosphate buffer covering a pH range of 6.0–7.0. The assay solution consisted of 25 mM buffer, 0.20 mM H_2_O_2_ and ~200 nM SaDyP1.

The Michaelis-Menten constant (*K*_m_) and catalytic constant (*k*_cat_) for ABTS and DMP were calculated in substrate-dependent enzymatic assay. The assay solution consisted of 25 mM citrate buffer (pH 4.5 for Acid Blue 324 and pH 4.0 for other dyes), 0.20 mM H_2_O_2_ and ~200 nM SaDyP and different concentrations of the substrates. The same conditions were measured three times, and the mean and standard deviation are shown. The data were fit to the Michaelis-Menten equation, Haldane equation or Hill equation. Kinetic parameters were calculated using molar absorption coefficients of ε_420_ = 36,000 cm^−1^M^−1^ for ABTS, ε_472_ = 49,600 cm^−1^M^−1^ for DMP, ε_608_ = 7700 cm^−1^M^−1^ for Acid Blue 324.

### 4.6. Decolorizing Rates for Anthraquinone and Azo Dyes

The ability of SaDyP1 to degrade representative dyes containing anthraquinone or azo chromophores was also examined. The assay solution consisted of 25 mM citrate buffer (pH 4.5 for Acid Blue 324 and pH 4.0 for other dyes), 0.20 mM H_2_O_2_ and ~200 nM SaDyP, additionally containing 0.20 mM Acid Blue 324 (ε_608_ = 7700 cm^−1^M^−1^), 0.10 mM AQ-2 (ε_600_ = 8300 cm^−1^M^−1^), 0.10 mM M303 (ε_476_ = 6200 cm^−1^M^−1^), 0.02 mM Reactive Black 5 (ε_598_ = 37,000 cm^−1^M^−1^) or 0.03 mM Reactive Red 33 (ε_500_ = 23,000 cm^−1^M^−1^). 

### 4.7. Culture and Fractionation of S. avermitilis

A suspension of *S. avermitilis* containing 2.0 × 10^9^ spores was inoculated into 200 mL YEME medium without sucrose and incubated for 20 h at 30 °C with shaking at 180 rpm. Mycelia from 30 mL cultures were collected by centrifugation at 7200× *g* for 30 min. Collected mycelia were disrupted three times at 2.0 × 10^4^ psi using a French press (Otake Seisakusyo) and centrifuged for 30 min at 7200× *g* to pellet cell debris. The precipitates were suspended in 1 mL of phosphate buffer and solubilized by incubating overnight with an equal volume of 10% SDS. An equal volume of 50% glycerol was added to the supernatant, and the total volume was concentrated ~40-fold by ultrafiltration (Amicon Ultra-15 30 K; Merck Millipore, Burlington, USA). Proteins in the concentrated supernatant were resolved by electrophoresis using 12 µL of supernatant per lane.

## Figures and Tables

**Table 1:** Peroxidase activity of SaDyP1 purified with or without hemin using ABTS as a substrate.

	Activity (μmol/min/mg)	Rz Value
hemin (-)	1.88	1.22
hemin (+)	1.74	1.57

**Table 2:** Dye-decolorizing activity of purified SaDyP1 toward anthraquinone or azo.

	Chromophore	ε at λ Max (nm)	Initial Conc. (μM)	Decolorizing Rate (μmol/min/mg)
Acid Blue 324	AQ ^a^	608	200	1.02
AQ-2	AQ	600	100	N.D. ^b^
M303	AQ	476	100	N.D.
Reactive Black 5	AZ	598	20	N.D.
Reactive Red 33	AZ	500	20	N.D.

^a^ AQ; anthraquinone, AZ; azo. ^b^ N.D.; not detected.

**Table 3:** Kinetic parameters of purified SaDyP1 toward several substrates.

	pH	*K*_m_ (mM)	*k*_cat_ (s^−1^)	*K*_i_ (mM)	*k*_cat_/*K*_m_ (M^−1^s^−1^)
ABTS	4.0	1.2	2.8	13	2.4 × 10^3^
DMP	4.0	0.61	0.23	-	3.8 × 10^2^
Acid Blue 324	4.5	0.049	0.79	-	1.6 × 10^4^

## Data Availability

Data sharing is not applicable to this article.
